# Simultaneous UV Spectrophotometric Estimation of Ambroxol Hydrochloride and Levocetirizine Dihydrochloride

**DOI:** 10.4103/0250-474X.41464

**Published:** 2008

**Authors:** S. Lakshmana Prabhu, A. A. Shirwaikar, Annie Shirwaikar, C. Dinesh Kumar, G. Aravind Kumar

**Affiliations:** Department of Pharmaceutical Quality Assurance, Manipal College of Pharmaceutical Sciences, Manipal–576 104, India; 1 Department of Pharmaceutics, Manipal College of Pharmaceutical Sciences, Manipal–576 104, India; 2 Department of Pharmacognosy, Manipal College of Pharmaceutical Sciences, Manipal–576 104, India

**Keywords:** Ambroxol hydrochloride, levocetirizine dihydrochloride, λ max, spectrophotometric method

## Abstract

A novel, simple, sensitive and rapid spectrophotometric method has been developed for simultaneous estimation of ambroxol hydrochloride and levocetirizine dihydrochloride. The method involved solving simultaneous equations based on measurement of absorbance at two wavelengths 242 nm and 231 nm, the γ max of ambroxol hydrochloride and levocetirizine dihydrochloride, respectively. Beer's law was obeyed in the concentration range 10–50 μg/ml and 8–24 μg/ml for ambroxol hydrochloride and levocetirizine dihydrochloride respectively. Results of the method were validated statistically and by recovery studies.

Ambroxol hydrochloride (AMB) is chemically, trans-4-((2-amino-3,5-dibromobenzyl) amino) cyclohexanol hydrochloride. Levocetirizine dihydrochloride (LEVC) is chemically, (RS)-2-{4-[(R)-p-chloro-α-phenylbenzyl]-1-piperazinyl} ethoxyacetic acid dihydrochloride^1^. AMB reduces bronchial hyper-reactivity and acts as a mucolytic and cough suppressant^1^. LEVC is usually used in allergic conditions including rhinitis^1^. Combination of AMB and LEVC is used for the treatment of bronchitis. These two drugs are not official in any pharmacopoeia; hence no official method is available for the simultaneous estimation of AMB and LEVC in formulations. Capillary electrophoresis^2^–^4^, spectrometry^5^, gas chromatography^6^,^7^, LC with potentiometric detection^8^, MS detection^9^ and UV detection^10^–^13^ methods have been reported for the estimation of AMB. However, no references have been found for simultaneous determination of AMB and LEVC in pharmaceutical formulations. A successful attempt has been made to estimate these two drugs simultaneously by spectrophotometric analysis.

A Shimadzu UV/Vis spectrophotometer, model-1601 (Japan) was employed with spectral bandwidth of 0.1 nm and a wavelength accuracy of ±0.5 nm with automatic wavelength correction with a pair of 3 mm quartz cells. AMB and LEVC (Aristo Pharma Ltd.), methanol (Merck India Ltd., Mumbai) and distilled water were used in the present study.

Stock solutions (500 μg/ml) of AMB and LEVC were prepared by dissolving separately in 20 ml of water in a 100 ml clean volumetric flask, and the volume was made up to 100 ml with distilled water. The maximum absorbance of AMB and LEVC was obtained at 244 nm (λ_2_) and 231 nm (λ_1_), respectively. AMB and LEVC showed linearity with absorbance in the range of 10–50 μg/ml and 8-24 μg/ml at their respective maxima, which were validated by least square method. Coefficients of correlation were found to be 0.9992 for AMB and 0.9993 for LEVC. For simultaneous estimation of AMB and LEVC, a series of standard solutions in concentration range of 2 to 24 μg/ml, were prepared by diluting appropriate volumes of the standard stock solutions. The scanning of solutions of AMB and LEVC were carried out in the range of 200 to 400 nm against water as blank for obtaining the overlain spectra that are used in the analysis (fig. 1). Absorbance and absorptivities of series of standard solutions were recorded at selected wavelengths λ_1_ and λ_2_.

**Fig. 1:**
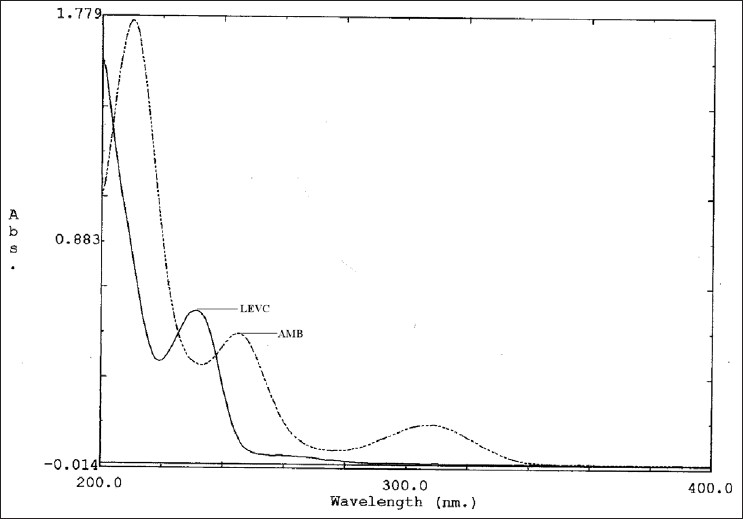
Overlain spectra of AMB and LEVC. Overlain spectra of ambroxol hydrochloride (AMB) and levocetiricine dihydrochloride (LEVC) in water. X axis depicts wavelength and Y axis depicts absorbance.

The absorptivity values for AMB and LEVC are shown in Table 1. The optical characteristics and regression values for the calibration curve are presented in Table 2. The method employed simultaneous equations using Cramer's rule and matrices (C_1_=λ_2_ε_2_×Aλ_1_-λ_1_ε_2_×Aλ_2_/λ_1_ε_1_×λ_2_ε_2_-λ_1_ε_2_×λ_2_ε_1_ and C_2_=λ_1_ε_1_×Aλ_2_-λ_2_ε_1_×Aλ_1_/λ_1_ε_1_×λ_2_ε_2_-λ_1_ε_2_×λ_2_ε_1_). A set of two simultaneous equations were framed using the mean of absorptivity values, given as Aλ_1_ = 211 C_1_+312 C_2_ and Aλ_2_ = 263 C_1_+71 C_2_, where, C_1_ and C_2_ are the concentrations of AMB and LEVC, respectively in simple solution (μg/ml). Aλ_1_ and Aλ_2_ are the absorbance of the sample solution measured at 231 and 244 nm, respectively.

**TABLE 1:** ABSORPTIVITY VALUES FOR AMBROXOL HYDROCHLORIDE AND LEVOCETRIZINE DIHYDROCHLORIDE

Concentration (μg/ml)		Absorptivity			
	
AMB	LEVC	231 nm		244 nm	
			
		AMB	LEVC	AMB	LEVC
2	2	210	310	261	70
4	4	212	311	261	73
6	6	212	314	264	72
8	8	209	312	262	71
10	10	208	312	263	71
12	12	211	313	263	70
14	14	212	310	261	72
16	16	212	312	262	70
20	20	212	311	265	71
24	24	211	310	262	71
	Mean	211	312	263	71
	SD	1.45	1.35	1.27	0.99

AMB and LEVC stands for ambroxol hydrochloride and levocetricine dihydrochloride, respectively

**TABLE 2:** REGRESSION AND OPTICAL CHARACTERISTICS OF AMBROXOL HYDROCHLORIDE AND LEVOCETIRIZINE DIHYDROCHLORIDE

Parameters	AMB	LEVC
λ_max_	244 nm	231 nm
Beer's Law range	10-50 μg/ml	8-24 μg/ml
Molar Absorptivity (0.001 absorbance unit/mole. cm/dm^3^)	9.944×10^3^	1.4409×10^4^
Sandell's sensitivity (μg/cm^2^/0.001 absorbance unit)	0.0379	0.0321
Regression values:		
Slope	0.0262	0.0302
Intercept	+0.0002	+0.008
Regression coefficient	0.9992	0.9993

AMB and LEVC stands for ambroxol hydrochloride and levocetricine dihydrochloride, respectively

Twenty tablets were weighed accurately. The average weight was determined and then ground to a fine powder. A quantity equivalent to 75 mg of AMB and 5 mg of LEVC were transferred to a 100 ml volumetric flask. The contents were sonicated for 10 min with 50 ml of distilled water and the volume was made up with distilled water. The solution was then filtered through a Whatman filter paper No. 40. The solution was further diluted with distilled water, to give concentrations of 30 and 2 μg/ml of AMB and LEVC, respectively. The absorbance of the resulting solution was measured at 231 and 244 nm.

To study accuracy, reproducibility, and precision of the proposed methods, recovery studies were carried out at three different levels by addition of standard drug solution to preanalysed samples. Results of recovery studies were found to be satisfactory which are presented in Table 3.

**TABLE 3:** RECOVERY STUDIES ON AMBROXOL HYDROCHLORIDE AND LEVOCETIRIZINE DIHYDROCHLORIDE IN SYNTHETIC MIXTURE

Drug in standard mixture solution (μg/ml)		% Recovery		Coefficient of variance (%)	
		
AMB	LEVC	AMB	LEVC	AMB	LEVC
2	2	99.28±0.341	98.88±0.555	0.311	0.491
4	4	99.52±0.254	99.42±0.308	0.209	0.256
8	6	99.13±0.205	99.03±0.404	0.322	0.460

AMB and LEVC stands for ambroxol hydrochloride and levocetricine dihydrochloride, respectively. The results are mean of three readings (n=3). % Recovery is expressed as mean ± standard deviation

The proposed method for simultaneous estimation of AMB and LEVC in combined sample solutions was found to be simple, accurate and reproducible. Beer's law was obeyed in the concentration range of 10-50 μg/ml and 8-24 μg/ml for AMB and LEVC, respectively. Co-efficient of variation was found to be 0.9992 and 0.9993 for AMB and LEVC, respectively. The percentage recovery studies were found to be in the range of 99.13 to 99.52% and 98.88 to 99.42% for AMB and LEVC, respectively. Once the equations are determined, analysis requires only the measuring of the absorbance of the sample solution at two wavelengths selected, followed by a few simple calculations. It is a method that can be employed for routine analysis in quality control laboratories.
